# Pharmacokinetics Alterations of Midazolam Infusion versus Bolus Administration in Mechanically Ventilated Critically Ill Patients

**Published:** 2013

**Authors:** Mohammad Taghi Beigmohammadi, Majid Hanifeh, Mohammad Reza Rouini, Behjat Sheikholeslami, Mojtaba Mojtahedzadeh

**Affiliations:** a *General ICU Department, Imam Khomeini University Hospital, Tehran University of Medical Sciences, Tehran, Iran. *; b *Department of Clinical Pharmacy, School of Pharmacy, Tehran University of Medical Sciences, Tehran, Iran.*; c *Department of Pharmaceutics, Pharmaceutical Sciences Research Center. School of Pharmacy, Tehran University of Medical Science, Tehran, Iran. *

**Keywords:** Midazolam, Mechanical ventilation, Pharmacokinetics, Continuous infusion, Intermittent bolus doses

## Abstract

There is no randomized study carried out in order to compare their pharmacokinetic parameters although midazolam, as a sedative, has been widely administered via continuous infusion as well as intermittent bolus doses in mechanically ventilated critically ill patients. We prospectively investigated the effect of these two principal methods on pharmacokinetic parameters in 23 of mentioned patients (16 males, 7 females) with the mean (± SD) age of 41.22 ± 17.5. All patients received total dose of 72 mg throughout the test days, 9 of whom received 1 mg/h (continuous infusion) and the rest obtained 4 mg / 4 h (intermittent bolus doses). Blood samples were collected at 8 and 4 h prior to the end time of drug administration (zero time), zero time and 4, 8, 12, 20 and 30 h after it. APACHE (Acute Physiology and Chronic Health Evaluation) II required data was recorded daily and the patients’ mean score was 16.26 ± 4.38. The mean (± SD) value of pharmacokinetic parameters of Midazolam in continuous infusion and intermittent bolus doses methods were as follows: (t½ = 17.88 ± 14.65 h, Cl = 21.80 ± 14.95 L/h) vs. (t½ = 19.74 ± 12.45 h, Cl = 29.43 ± 19.45 L/h). Volume of distribution (Vd) was measured in continuous infusion group which was 612.58 ± 582.93 L. The calculated clearance and half-life were found not to be significantly different (p < 0.05). The patients might be exposed to similar undesired effects due to the large volumes of distribution following the administration methods studied.

## Introduction

Sedation is an important component of the treatment of mechanically ventilated critically ill patients (1). The majority of mechanically ventilated patients within the ICU receives sedative drugs to decrease anxiety, ensure comfort and facilitate treatments (2). Benzodiazepines, as a class, have been the sedatives of choice in ICUs worldwide since the early 1990s (3). Because of its rapid onset and short duration of action, low incidence of thrombophlebitis and pain in injection and minimal cardiovascular and respiratory effects, midazolam is readily distinguished from other benzodiazepines (4). 

Midazolam is 94 to 98% protein bound, has a short distribution t_1/2_ (is distributed quickly into the CNS), a large steady state V_d_ (V_ss_) [0.68 to 1.77 L/Kg], an intermediate plasma body total clearance (Cl) [18 to 39 L/h ] and a short elimination half-life (t_1/2 z_) (1.5 to 5 h) (5, 6).

In critically ill patients, alterations in plasma protein binding and the presence of any multi-organ disease result in a decreased elimination and increased V_d_ of midazolam. However, in ICU patients without significant end organ disease, midazolam clearance does not appear to be decreased (6).

In intensive care units, sedatives are often infused continuously. As compared with intermittent multiple dose boluses, this approach provides a more constant level of sedation and may increase the patient’s comfort (7). Furthermore, intermittent dosing of sedative medication may consume more nursing resources and detract from other aspects of patient care (8). Recent studies, however, have cast doubt on the practice of continuous sedation. For example, a study by Kollef *et al. *reported that continuous intravenous sedation may be associated with the prolongation of mechanical ventilation (9).

Consequently, it has been tried in this study to find the pharmacokinetic key parameters of midazolam following a continuous infusion versus the intravenous (IV) multiple dose boluses in mechanically ventilated critically ill patients.

## Experimental

*Patients*

The trial was performed on 23 mechanically ventilated critically ill patients, aged 18 to 65 years old, who admitted in Imam Khomeini Hospital General ICU Department, Tehran, Iran, between August 2008 and June 2011.

Patients with hepatic or renal failure, MAP < 65 mmHg, Platelets number < 100000, Serum Alb < 2.5 g/dL, Peep > 10 mmHg and seizure history were excluded from this study. Initial demographic data (Age, Sex, Diagnosis, and Possible Comorbidities) for each patient were recorded and Acute Physiology and Chronic Health Evaluation (APACHE II) was determined on a daily basis.

Having been approved by the Institutional Review Board for Human Study and Ethics Committee, Tehran University of Medical Sciences, in conformation with the principles in the Helsinki Declaration, a written informed consent was obtained from all eligible patients prior to the study interventions performing. All included patients were informed about the aim and risks of the study by the clinical investigators and they participated on the self-decision.

*Study protocol and drug administration*

The patients were divided to intermittent multiple dose boluses and continuous infusion groups, according to their randomly case numbers. Patients received continuous IV infusion of 1 mg/h (Group I, n = 9), or multiple dose IV boluses of 4 mg / 4 h Midazolam (Group II, n = 14) for 72 h. In case of insufficient analgesia, morphine was administered for break through pain (as 5 mg PRN). One-milliliter blood samples were collected in heparinized glass tubes at 8 and 4 h before the end time of drug administration (which was considered as Zero time: 0), after 0, 4, 8, 12, 20 and 30 h. For both groups, blood samples were obtained at the same time points. Plasma was separated from blood samples by centrifugation (10,000 g for 10 min) and stored at - 20ºC until analysis.

*Drug analysis*

Plasma concentration of Midazolam (ng/mL) was determined by the following HPLC method. To a 250 μL of plasma sample, 50 μL oxazepam (150 ng/mL) as internal standard and 50 μL NaOH (1N) were added. After mixing, samples were extracted with 1200 μL of ethyl acetate. After agitation (10 min) and centrifugation (10,000 g for 10 min), the organic layer was transferred into a conical tube glasses. After that, the organic phase was evaporated under a gentle air stream and reconstituted in 150 μL of mobile phase. A 100 μL aliquot of it was injected on to the HPLC system, consisting of a low-pressure gradient HPLC pump, a UV detector [wavelength set at 220 nm] and an online degasser, all from Knauer (Berlin, Germany). Separation was achieved by a Chromolith Performance RP-18e 100 mm × 4.6 mm column (Merck, Darmstadt, Germany) protected by a Chromolith guard cartridge RP-18e 5 mm × 4.6 mm. A mixture of acetonitrile-phosphate buffer 0.05 M (30:70, v/v) adjusted to pH of 4.1 by phosphoric acid at flow rate of 2 mL/min was used as mobile phase. The data were acquired and processed by means of ChromGate chromatography software (Knauer, Berlin, Germany).

*Pharmacokinetic calculations*

Elimination rate constant (K_e_) for midazolam was calculated from the blood samples obtained in zero time and after it. Midazolam elimination half-life (T_1/2_) was obtained from 0.693/K_e_. C_ss_ was calculated for group I using the equation of C_p_ = C_ss_ (1 - e^-kt^). Clearance was calculated using the equation of Clearance = Infusion rate (K_0_) /Css. Volume of distribution was obtained from Vd = Cl / K_e_.

*Statistical analysis*

Data were presented as mean ± SD and analyzed using independent t-test. Fisher Exact test or Mann-Whitney U-test was employed to compare demographic data. Correlations between pharmacokinetic parameters and physiologic indices were investigated using Pearson’s test. All statistical analyses were performed by Statistical Package for Social Science version 16 (SPSS Inc., Chicago, IL, USA). Probability values of p < 0.05 were considered statistically significant.

## Results and Discussion

Mechanically ventilated critically ill patients confront major stress align with their acute medical problem. Non-pharmacologic treatment such as relaxation in bed and verbal confidence should be initially considered but sedatives and analgesics are usually required to make the ICU environment more endurable (10). If the pharmacokinetic changes of these drugs are well recognized in critically ill patients, they will be more properly administered in ICU (4). To achieve this goal, we have compared two common routes of midazolam administration in mentioned acutely ill patients.

There is a great concern about accumulation of midazolam in peripheral body tissues after long periods of drug administration (> 48 h) (11) and the main focus of this study is on the final elimination phase of midazolam after 72 h.

A total of 23 patients were enrolled in the study; 9 were randomly assigned to the infusion group (Group I) and 14 to the other one (Group II). The demographic characteristics and APACHE II daily scores were similar in both groups (Table 1).

**Table 1:** The demographic data of patients

	**Intermittent Bolus Doses (mean ± SD)**	**Continuous Infusion (mean ± SD)**	**p-Value**
Sex (male : female)	(1 : 0.5)	(1 : 0.4)	1.000 ( Fisher Exact test)
Age	45.21 ± 20.18	36.56 ± 15.88	0.33 (Mann-Whitney U-test )
Day 1 APACHE II	16.07 ± 4.58	15.22 ± 6.76	0.72
Day 2 APACHE II	16.71 ± 4.84	14.11 ± 5.67	0.25
Day 3 APACHE II	16.29 ± 4.14	13.11 ± 4.43	0.10
Day 4 APACHE II	18.71 ± 6.13	13.56 ± 4.80	0.05
Day 5 APACHE II	18.14 ± 5.91	15.13 ± 4.70	0.23

**Figure 1:**
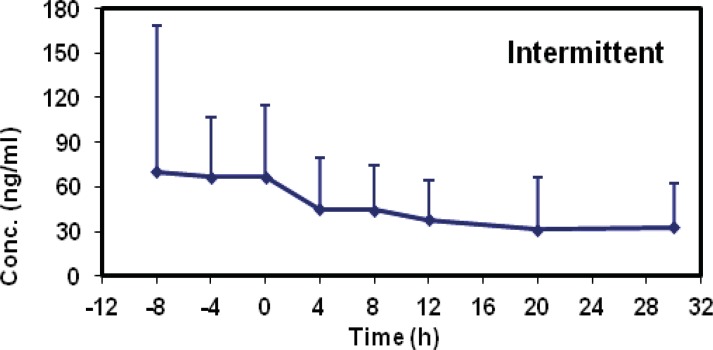
Concentration curve for patients in intermittent bolus doses group

**Figure 2:**
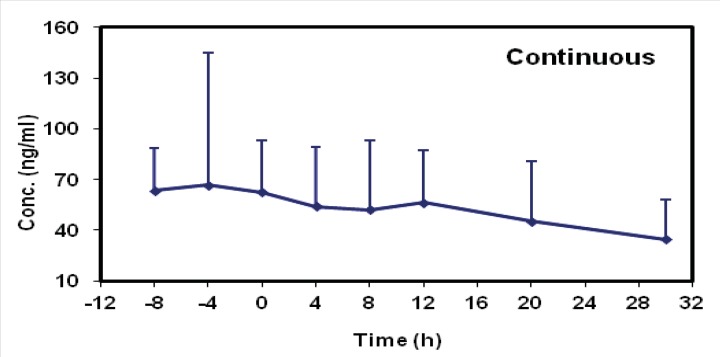
Time-concentration curve for patients in continuous infusion group

The mean concentrations of midazolam in sampling times followed by two methods are shown in Table 2 and the time-concentration curve of each method is shown in Figures 1 and 2.

**Table 2:** The mean concentrations of Midazolam (ng/mL) in sampling times following two methods.

	**Intermittent Bolus Doses (mean ± SD)**	**Continuous Infusion (mean ± SD)**	**p-Value**
C_p64_ (-8)	76.36 ± 111.39	60.11 ± 29.96	0.71
C_p68_ (-4)	69.48 ± 41.45	71.90 ± 94.45	0.95
C_p72_ (0)	69.49 ± 49.60	60.83 ± 37.71	0.66
C_p76_ (+4)	43.29 ± 35.63	53.7 ± 38.87	0.52
C_p80_ (+8)	41.18 ± 31.29	38.91 ± 33.6	0.88
C_p84_ (+12)	31.46 ± 22.75	46.90 ± 35.66	0.29
C_p92_ (+20)	26.19 ± 33.17	36.63 ± 37.01	0.55
C_p102 _(+30)	25.85 ± 25.90	20.86 ± 18.05	0.71

Midazolam pharmacokinetic parameters in each group were summarized in Table 3. 

**Table 3:** Midazolam pharmacokinetic parameters of patients following two methods

	**Intermittent Bolus Doses (mean ± SD)**	**Continuous Infusion (mean ± SD)**
Half-life (h)	19.74 ± 12.45	17.88 ± 14.65
Clearance (Lit/h)	29.43 ± 19.45	21.80 ± 14.95
V_d_ (Lit)	-	612.58 ± 582.93
C_ss_ (ng/mL)	-	69.44± 44.94

The mean elimination half-life values were 17.88 ± 14.65 (group I) and 19.74 ± 12.45 (group II). There was no statistical difference between the two methods (p = 0.207; CI 0.95: - 4.54, 19.69). The mean clearance value of midazolam was decreased in group I (21.80 ± 14.95) as compared with group II (29.43 ± 19.45) but its amount was not statistically significant and they were similar in both groups (p = 0.757, CI 0.95: - 19.79, 14.60).

Midazolam pharmacokinetic parameters in each group were summarized in Table 3. The mean elimination half-life values were 17.88 ± 14.65 (group I) and 19.74 ± 12.45 (group II). There was no statistical difference between the two methods (p = 0.207; CI 0.95: - 4.54, 19.69). The mean clearance value of midazolam was decreased in group I (21.80 ± 14.95) as compared with group II (29.43 ± 19.45) but its amount was not statistically significant and they were similar in both groups (p = 0.757, CI 0.95: - 19.79, 14.60).

The first remarkable finding in this study like other pervious similar trials (12-14) was the significant standard deviation with respect to average which indicates the wide interpatient variability of midazolam pharmacokinetic parameters. This phenomenon was also seen with steady state concentrations (C_ss_) of midazolam which complicates its kinetic study in these patients.

Although the mean elimination half-life values following the both methods were similar, they were more than three times longer in comparison with normal volunteers (5).

The elimination half-life of the drug is calculated by the equation: (elimination half- life = 0.7 × distribution volume / clearance) (13). Prolongation of midazolam elimination half-life seems to be related to a decrease in clearance or an increase in volume of distribution (Or both of them).

Mechanical ventilation with or without PEEP (Positive End Expiratory Pressure) can decrease the cardiac output, liver and kidney blood flow, glomerular filtration and urine output (15). In theory, these hemodynamic alterations are able to reduce the clearance of several drugs especially those mainly eliminated by liver (16). This theory was applied in previous studies on mechanically ventilated critically ill patients even by drugs with low hepatic extraction ratio (*e.g. *theophylline (17), aminophylline (18) and lorazepam (14)). So we expected a decrease in midazolam clearance, which has intermediate to high hepatic extraction ratio, in the mentioned patients. But in the present study, the mean clearance values for both groups were fall in normal range (5). This result might be due to a possible optimization of our hemodynamic profile and ventilator indices. So we surveyed the relationship between physiologic parameters (APACHE II score, HR, MAP, GCS) and pharmacokinetic data (clearance, half-life). There was only a poor direct correlation between the APACHE II score and half-life (r^2^ = 0.4, p = 0.058).

Interestingly, the elimination half-life prolongation in our study seems to be the result of an increased volume of distribution which is supported by calculated data following continuous infusion method. Taking the exclusion criteria of this study into consideration, the increase in volumes of distribution could be related to series of factors such as fluid shifts, pH changes, drug interactions (19), protein binding (20), tissue perfusion and permeability derangements (21). Apart from this, V_d_ remained high during the chronic phase and following the first 72 h of hemodynamic stabilization which seems to be the most valuable finding of this study. It is more likely because of the alterations in microcirculation and cytopathic hypoxia. With regard to our graphs shown in the results, a long time should be needed for drug clearance from body following each of two methods. Therefore, it is expected to see the adverse effects of midazolam accumulation even by intermittent multiple dose boluses. These findings may be translated into the variety of complications such as delirium, longer periods for mechanical ventilation and prolonged ICU stay (22).

In this study, we have tried to minimize the confounding factors by our exclusion criteria which have limited the number of patients qualified for our trial. Geriatric (≥ 65) and pediatric (≤ 18) patients as well as the patients with hepatic or renal failure, MAP < 65 mmHg, Platelets number < 100000, Serum Alb < 2.5 g/dL, PEEP > 10 mmHg and seizure history were excluded from this study.

Larger volumes of distribution and accumulation of midazolam in peripheral body tissues have been the most considerable issue in these pharmacokinetic studies (11).So, safer alternatives with more predictable pharmacokinetic profile (new *α*_2_ agonists) and as needed (PRN) orders for midazolam might be considered for more rational patient care.

In conclusion, there is no significant pharmacokinetic difference between the two methods (1 mg/h versus 4 mg / 4 h) of midazolam administration in studied patients and they might be exposed to similar undesired effects due to the large volumes of distribution following drug administration. These results direct us to put longer break periods (> 4 h) as long as midazolam is administered via intermittent bolus doses or to interrupt its daily sedative infusion to prevent the adverse effects. The continuous infusion method would be the preferable one due to its ease of administration, constant level of sedation and more hemodynamic stability in the same setting. 
